# Diseases of the Skin

**Published:** 1863

**Authors:** 


DISEASES OF THE SKIN: By ERASMUS WILSON, F.R.S. Fifth American, from the fifth and revised London edition, with plates and illustrations on wood. PHILADELPHIA: BLANCHARD & LEA. 1863.

This is a new edition of a well-known work, which has been before the profession for twenty years. It is worthy of the careful study of every student, and will be found profitable in the library of every practitioner.

In this edition, the American publishers have very properly added to the plates those prepared by Mr. Wilson, to illustrate his work on "Constitutional Syphilis and Syphilitic Eruptions." The volume contains 694 pages and is published in a good style.

For sale by W. B. Keen & Co., Booksellers, Chicago.

